# Can we achieve better trial recruitment by presenting patient information through multimedia? Meta-analysis of ‘studies within a trial’ (SWATs)

**DOI:** 10.1186/s12916-023-03081-5

**Published:** 2023-11-08

**Authors:** Vichithranie W. Madurasinghe, Peter Knapp, Sandra Eldridge, David Collier, Shaun Treweek, Jo Rick, Jonathan Graffy, Adwoa Parker, Chris Salisbury, David Torgerson, Kate Jolly, Manbinder S. Sidhu, Christopher Fife-Schaw, Mark A. Hull, Kirsty Sprange, Elizabeth Brettell, Sunil Bhandari, Alan Montgomery, Peter Bower

**Affiliations:** 1 Nuffield Department of Population Health, University of Oxford, Richard Doll Building, Old Road Campus, Roosevelt Drive, Oxford, OX3 7LF UK; 2 Department of Health Sciences, University of York & the Hull York Medical School, York, YO10 5DD UK; 3 Centre for Clinical Trials and Methodology, Institute of Population Health Sciences, Queen Mary University of London, 58 Turner Street, London, E1 2AB UK; 4 Barts NIHR Biomedical Research Centre, William Harvey Research Institute, Queen Mary University of London, London, EC1M 6BQ UK; 5 Health Services Research Unit, University of Aberdeen, 3Rd Floor, Health Sciences Building, Foresterhill, Aberdeen, AB25 2ZD UK; 6 National Institute of Health Research School for Primary Care Research, Manchester Academic Health Science Centre, Centre for Primary Care, University of Manchester, Oxford Road, Manchester, M13 9PL UK; 7 General Practitioner Arbury Road Surgery 114, Arbury Road, Cambridge, CB4 2JG UK; 8 York Trials Unit, Department of Health Sciences, University of York, York, YO10 5DD UK; 9 Centre for Academic Primary Care, Department of Population Health Sciences, Bristol Medical School, University of Bristol, Canynge Hall, 39 Whatley Road, Bristol, BS8 2PS UK; 10 Department of Health Sciences, University of York, Heslington, York YO10 5DD UK; 11 Institute of Applied Health Research, University of Birmingham, Edgbaston, Birmingham, B15 2TT UK; 12 Health Services Management Centre, School of Social Policy, University of Birmingham, Edgbaston, Birmingham, B15 2RT UK; 13 University of Surrey, Guildford, UK; 14 Leeds Institute of Medical Research, University of Leeds, Leeds, LS9 7TF UK; 15 Nottingham Clinical Trials Unit, University of Nottingham, Nottingham, NG7 2RD UK; 16 Birmingham Clinical Trials Unit, University of Birmingham, Birmingham, B15 2TT UK; 17 Department of Renal Medicine, Hull University Teaching Hospitals NHS Trust, and Hull York Medical School, Hull, East Yorkshire HU3 2JZ UK; 18 NIHR School for Primary Care Research, School of Health Sciences, Manchester Academic Health Science Centre, University of Manchester, Manchester, M13 9PL UK

**Keywords:** Recruitment, Information, User testing, Research methodology, Randomised controlled trial, SWATs, Meta-analysis

## Abstract

**Background:**

People need high-quality information to make decisions about research participation. Providing information in written format alone is conventional but may not be the most effective and acceptable approach. We developed a structure for the presentation of information using multimedia which included generic and trial-specific content. Our aim was to embed ‘Studies Within A Trial’ (SWATs) across multiple ongoing trials to test whether multimedia presentation of patient information led to better rates of recruitment.

**Methods:**

Five trials included a SWAT and randomised their participants to receive a multimedia presentation alongside standard information, or standard written information alone. We collected data on trial recruitment, acceptance and retention and analysed the pooled results using random effects meta-analysis, with the primary outcome defined as the proportion of participants randomised following an invitation to take part.

**Results:**

Five SWATs provided data on the primary outcome of proportion of participants randomised. Multimedia alongside written information results in little or no difference in recruitment rates (pooled odds ratio = 0.96, 95% CI: 0.79 to 1.17, *p*-value = 0.671, *I*^2^ = 0%). There was no effect on any other outcomes.

**Conclusions:**

Multimedia alongside written information did not improve trial recruitment rates.

**Trial registration:**

ISRCTN71952900, ISRCTN 06710391, ISRCTN 17160087, ISRCTN05926847, ISRCTN62869767.

**Supplementary Information:**

The online version contains supplementary material available at 10.1186/s12916-023-03081-5.

## Background

Trials remain critical to evidence-based practice, but recruitment remains a significant challenge worldwide [1, 2]. Despite these challenges, the evidence base concerning effective recruitment strategies is weak, with the Cochrane review in this area reporting only 3 strategies with ‘high certainty evidence’ [3].

To rapidly develop the evidence base, a model has developed of testing promising recruitment strategies by embedding randomised tests in multiple ongoing trials (so-called co-ordinated ‘Studies Within a Trial’ or SWAT). This model has been used by the START [4], TRECA [5] and PROMETHEUS [6] research programmes and has contributed to major growth in the evidence base for recruitment and retention. One of the innovations tested by both the START and TRECA programmes was the potential for multimedia to provide improved information to trial participants and potentially enhance the likelihood that they would be successfully recruited to a trial.

### The potential role of multimedia

Written information in trials has been criticised for length and complexity and a lack of clear structure to help patients find the information they need. Changes based on user testing and information design can produce information sheets that are easier for patients to understand [7–9], although our previous programme of co-ordinated SWATs found that such changes did not lead to improvements in recruitment [10].

Provision of audio-visual information about trials may be another way to improve the delivery of information and enhance patient decision-making. Previous studies suggest that audio-visual presentation leads to a small increase in patient understanding, but may have less effect on recruitment [11]. Multimedia information is defined by its use of more than one format, and in terms of patient information has generally entailed the digital presentation of a combination of written text, recorded speech, pictograms and video (including scenario portrayal and animations). It can increase levels of user attention and engagement, not only through the choice of format, which allows users to ‘personalise’ or tailor the information, but also through dual channel stimulation and efficient cognitive demands [12]. It may better meet the needs of an audience increasingly accustomed to obtaining information digitally. There is some evidence that this can increase comprehension within the research consent process [13–15].

Reviews of the impact of multimedia interventions on research participation have explored a variety of outcomes, including knowledge and understanding, recall, willingness to participate, perceptions of the value of research and decision-making outcomes. Only a small number of studies explored the effects of multimedia materials in the Cochrane review on improving recruitment to trials [16] and the overall conclusion was of uncertainty concerning the effects. Given the limited evidence base, further research through co-ordinated SWATs is clearly warranted. Testing the same strategy across multiple trials provides the opportunity to produce both a more precise estimate of effect and some exploration of whether effects vary by trial type. In this paper, we synthesise SWATs of multimedia use to test their effects on recruitment.

## Methods

START was a programme of methodology research funded by the Medical Research Council, which aimed to (a) develop methods for design, delivery and reporting of SWATs [17] and (b) to deliver two sets of co-ordinated SWATs testing two recruitment interventions across multiple trials. The methods of the START programme have been published [4], followed by the first co-ordinated set of SWATs testing the effects of written information sheets optimised through user testing [10]. The current paper represents the second set of SWATs.

To recruit studies for each set of SWATs, we contacted chief investigators through the National Institute of Health and Care Research (NIHR) Health Technology Assessment and Efficacy and Mechanism Evaluation programmes or the Primary Care Research Network, selecting trials with at least 800 participants to be approached for recruitment where the design was amenable to the multimedia recruitment model. We aimed to recruit 6 host trials in which to embed our multimedia intervention.

### Development of the multimedia intervention

Multimedia content was generated by team members, informed by a review of factors identified by patients as determinants of decisions about trial participation [18], as well as input from patient and public involvement (PPI) contributors and qualitative experts on patient health experiences.

Content included *study-specific information* (e.g. study purpose, risks) and *generic information* (e.g. confidentiality). PPI contributors and qualitative experts developed study-specific components involving bespoke themes such as investigator details and benefits of participation. Generic information components included information on informed consent, randomisation and confidentiality. Existing video clips of patients discussing their experiences of participation were edited for length and carefully matched to these components. Additional file 1 provides an example of the range of material presented, with text and video material in separate tabs for the particular trial (for example, information about this study’, ‘what will happen during the study’) and trials in general (‘why get involved’, ‘leaving a study’). The material was designed to provide a much more flexible set of options for patients in terms of how much information they accessed, and in what order, as well as being designed to be more accessible and engaging. The multimedia intervention was developed by a commercial company for use on a range of platforms including desktops and smartphones.

Access to the multimedia resource was provided within the patient information sheet, with a URL link and QR code to assist with easy access (see Additional file 2 for the presentation of the resource to patients). Although we randomised participants to access the multimedia, it was entirely the choice of the participant whether they actually engaged with the multimedia information (alongside the written information) as part of their decision-making process about the trial.

### Methods of the SWAT

In each SWAT participants being approached to take part were randomised to receive the multimedia alongside written information or standard written information alone. Individual randomisation was used where possible to maximise power and precision and minimise selection bias, but we adopted cluster randomisation where preferred by the host trial for logistical reasons.

### Outcome measures

The primary outcome was recruitment, defined as the proportion of participants recruited and randomised to a host trial following an invitation to take part. The denominator for the outcome was the total number of potentially eligible participants offered entry to the trial. Depending on the particular trial, this would include a mix of eligible and ineligible patients according to the formal inclusion and exclusion criteria.

Secondary outcomes were:

1. Acceptance, defined as the proportion of potentially eligible participants who express interest in participating (i.e. posting a reply or attending a recruitment appointment). We anticipated that in some SWATs, the number of participants recruited to the host trial could be different from numbers of participants responding positively, due to eligibility criteria used in the host trial.
2. Retention, defined as the proportion retained at primary outcome measurement endpoint of the host trial.

### Ethical approval

START was approved by the National Research Ethics Service (NRES) Committee, Yorkshire and the Humber – South Yorkshire (Ref: 11/YH/0271) on the 5 August 2011. Each individual host trial had its own ethical agreement and registration.

### Data analysis

Analyses of recruitment were conducted according to a statistical analysis plan. Outcomes were first described separately by study arm and then compared using logistic regression to estimate the between-group odds ratio and corresponding 95% confidence interval. The data from each SWAT were meta-analysed using the Stata *metan* command (Stata version 14.2) using random effects models based on likely clinical and methodological heterogeneity. Statistical inconsistency was quantified using the *I*^2^ statistic. In the meta-analysis, we used a two-stage strategy where each individual SWAT was analysed using the appropriate analysis methods (i.e. taking into account whether it was individually or cluster randomised) to generate trial-level summary statistics (e.g. odds ratios) first, and then the results from individual SWATs were combined across trials using the Stata *metan* command (Stata version 14.2).

We performed pre-specified subgroup analyses investigating differences between studies based on underlying recruitment rates (low defined as a recruitment rate of 5% or below in control group vs. higher rates). We hypothesised that when the baseline recruitment rate is low, the increase in the absolute recruitment rate associated with a recruitment intervention may be higher. A second planned analysis comparing patients with a known diagnosis versus participants ‘at risk’ was not conducted as it proved difficult to assign trials to the categories reliably.

## Results

We originally recruited 6 trials for the SWATs. Only one trial has reported the individual SWAT evaluation [19]. Table 1 describes the characteristics of the host trials and the SWATs. One host trial was only able to report accurate data on 11/37 sites randomized [20] and was excluded from all the analyses (available data by arm are reported in Additional file 3). All host trials were individually randomised, but 2 SWATs used cluster randomisation (general practices, endoscopy units or week of recruitment) because this was operationally easier. One host trial included the same SWAT in two separate groups of practices [21], one group allocating patients on the basis of first contact letters and a second group allocating patients on reminder letters after they had initially been contacted.

**Table 1:** Trial characteristics

Trial name	Population	Host trial intervention and comparison	Design of the host trial	Design of SWAT
GHT2000 [21]	Inactive 18–74-year-olds with hypertension, suspected hypertension, pre-hypertension or high-normal blood pressure	Interventions: (i) GP gym-based referral plus web tool, (ii) sport referral (iii) sport referral plus web tool Comparator: GP gym-based referral	Individually randomised four-arm group trial	Two-arm trial, individually randomised
PSM COPD [22]	COPD patients aged 18 years or older with mild dyspnoea	Intervention: a telephone-based self-management intervention Comparator: usual care	Individually randomised two-arm group trial	Two-arm trial, clustered by general practice
HI-Light [23]	Patients aged 5 years and over with vitiligo	Interventions: (i) Handheld narrowband UVB (NB-UVB) and (ii) a combination of potent topical corticosteroid and NB-UVB, compared with potent topical corticosteroid	Individually randomised three-arm, placebo-controlled	Two-arm trial, individually randomised
seAFOod [24]	Patients aged 55–73 years identified during screening colonoscopy as being at ‘high risk’ for subsequent surveillance colonoscopy	Interventions: (i) 2 g eicosapentaenoic acid per day and (ii) 300 mg aspirin per day Comparator: placebo	Individually randomised 2 by 2 factorial trial	Two-arm trial, cluster randomised ( Endoscopy Unit taking part in the UK NHS Bowel Cancer Screening Programme)
STOP ACEi [20]	Patients with advanced progressive CKD receiving angiotensin-converting enzyme inhibitor or angiotensin receptor blockers (or both)	Intervention: Discontinue Angiotensin Converting enzyme inhibitor (ACEi)/Angiotensin Receptor Blocker (ARB) or combination of both Comparator: Continue ACEi, ARB or combination of both	Individually randomised two-arm group trial	Two-arm trial, individually randomised

**Fig. 1:**
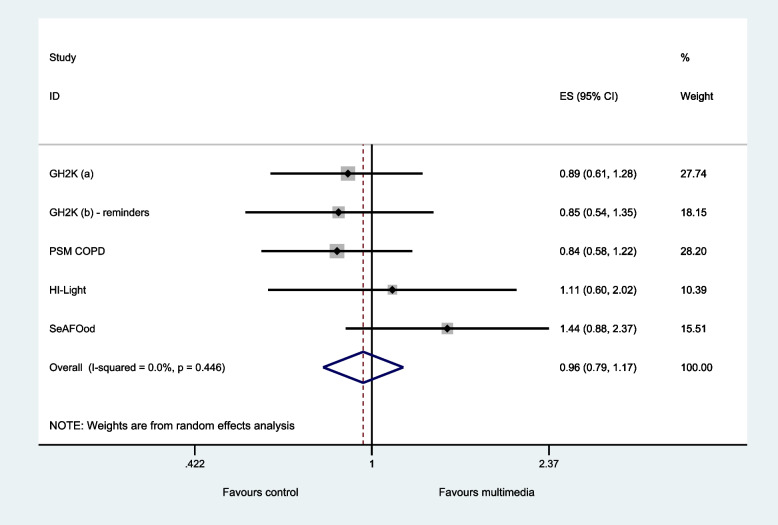
Primary outcome—randomised to host trial. Heterogeneity chi-squared = 3.72, *p* = 0.446; *I*.^2^ = 0.0%; test of pooled odds ratio = 1: *z* = 0.42, *p* = 0.671

Five host trials provided data on recruitment [21–24]. Access to multimedia resulted in little or no difference in recruitment rates (pooled odds ratio = 0.96, 95% CI: 0.79 to 1.17 *p*-value = 0.671, *I*^2^ = 0%) (Table 2 and Fig. 1).

**Table 2:** Primary outcome—randomised to host trial

Study	Standard	Multimedia	Odds ratio (95% CI)	% weight
GHT2000	64/1049 (5.9%)	57/1048 (5.4%)	0.89 (0.61 to 1.28)	27.7
GHT2000 (reminder)	41/1057 (3.9%)	35/1055 (3.3%)	0.85 (0.54 to 1.35)	18.2
PSM COPD	247/2280 (10.8%)	185/1934 (9.6%)	0.84 (0.58 to 1.22)	28.2
HI-Light	51/1136 (4.5%)	54/1094 (4.9%)	1.11 (0.60 to 2.02)	10.4
seAFOod	61/395 (15.4%)	68/333 (20.4%)	1.44 (0.88 to 2.37)	15.5
Pooled	464/5917 (7.8%)	399/5464 (7.3%)	0.96 (0.79 to 1.17)	100.0

**Fig. 2:**
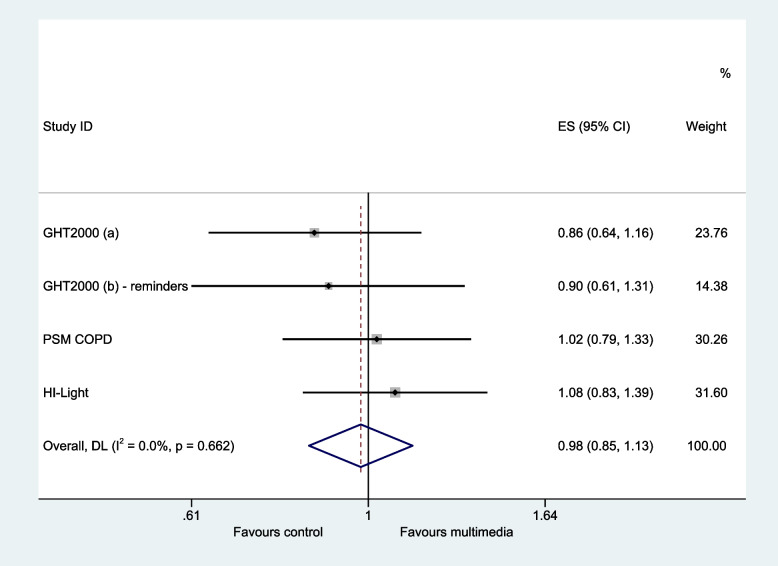
Secondary outcome—responded positively to invitation. Heterogeneity chi-squared = 1.59, *p* = 0.662; *I*.^2^ = 0.0%, test of pooled odds ratio = 1: *z* = 0.28, *p* = 0.778

Four host trials provided data on participant acceptance rates [21–23]. Access to multimedia resulted in little or no difference in the likelihood of responding positively to the invitation compared to participants receiving standard information (pooled odds ratio 0.98, 95% CI: 0.85 to 1.13, *p* value = 0.778, *I*^2^ = 0%) (Fig. 2 and Table 3).

**Table 3:** Secondary outcome—responded positively to invitation

Study	Standard	Multimedia	Odds ratio (95% CI)	% weight
GHT2000	100/1049 (9.5%)	87/1048 (8.3%)	0.86 (0.64 to 1.16)	23.8
GHT2000 (reminder)	59/1057 (5.6%)	53/1055 (5.0%)	0.90 (0.61 to 1.31)	14.4
PSM COPD	464/2280 (20.3%)	412/1934 (21.3%)	1.02 (0.79 to 1.33)	30.3
HI-Light	221/1136 (19.5%)	226/1094 (20.7%)	1.08 (0.83 to 1.40)	31.6
Pooled	844/5522 (15.3%)	778/5131 (15.2%)	0.98 (0.85 to 1.13)	100.0

**Fig. 3:**
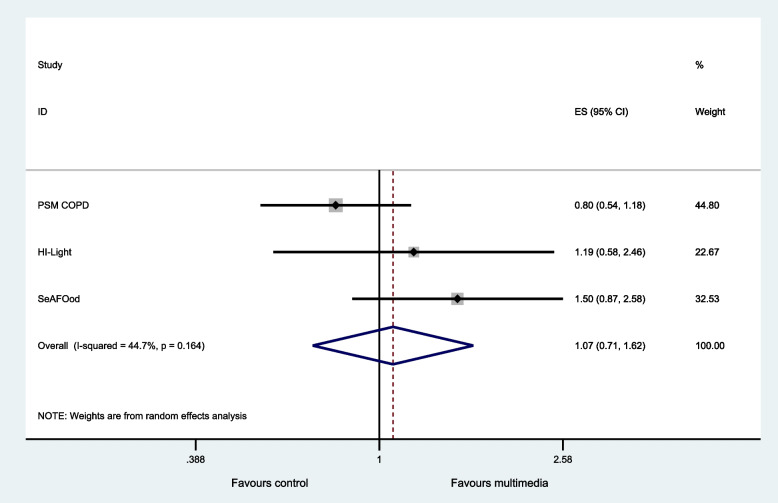
Secondary outcome—retained at primary endpoint of the host trial. Heterogeneity chi-squared = 3.62, *p* = 0.164; *I*.^2^ = 44.7%, test of pooled odds ratio = 1: *z* = 0.34, *p* = 0.737

Three SWATs provided data on retention [22–24]. Access to multimedia resulted in little or no difference in retention compared to participants receiving standard information (pooled odds ratio 1.07, 95% CI: 0.71 to 1.62, *p* value = 0.737, *I*^2^ = 44.7%) (Fig. 3 and Table 4).

**Table 4:** Secondary outcome—retained at primary endpoint of the host trial

Study	Standard	Multimedia	Odds ratio (95% CI)	% weight
PSM COPD (at 12 months)	223/2280 (9.8%)	159/1934 (8.2%)	0.80 (0.54 to 1.18)	44.8
HI-Light (at 9 months)	35/1136 (3.1%)	40/1094 (3.7%)	1.19 (0.58 to 2.46)	22.7
seAFOod (at 12 months)	49/395 (12.4%)	57/333 (17.1%)	1.50 (0.87 to 2.6)	32.5
Pooled	307/3811 (8.1%)	256/3361 (7.6%)	1.07 (0.71 to 1.62)	

There was no marked difference in intervention effects by baseline rate (odds ratio 0.94 95% CI 0.65 to 1.35 in low baseline trials compared with 0.99 95% CI 0.73 to 1.33 in high baseline trials).

## Discussion

### Summary

We tested the effects of access to multimedia information on trial recruitment and retention. In a number of SWAT evaluations that run through a diverse group of host trials, the intervention did not improve acceptance, recruitment or retention rates among participants.

### Strengths and limitations of the study

This programme of coordinated SWATs was one of the first to be initiated, demonstrating the broad feasibility of this strategy as a model for the more rapid development of an evidence base.

As with most SWATs, there was no formal sample size calculation for individual trials. The host trials undertaking the SWATs were self-selected and therefore the studies on which the programme was run represent a relatively specific (though diverse) group of study contexts, with most patients in the older age groups. It is possible that the variation in those contexts was sufficient to give the recruitment strategy a fair test across multiple designs and populations, and there was limited evidence of significant variation in effect. Even the pooled analysis of data from six trials left some imprecision in the estimate of effect. We have provided broad details of the patient populations sought for each of the trials, but we do not have detailed information on the demographics of those who took part in the SWATs. It is important to note that the participants in the SWATs are not the same as those taking part in the trials, as many people participate in the SWATs without entering the trial. Collecting such information is complicated and potentially burdensome for trial teams. One study was unable to provide the necessary data for our main analysis, which reduced the sample size available. In our experience, problems with delivering SWATs are fairly rare but these difficulties do highlight that SWATs can stretch the resources of already busy trial teams.

Although we planned to assess the use of the multimedia intervention, including its various elements, to provide better context to our outcome data, an error in the web-hosting software meant no accurate data on use were available. The SWATs only provided patients with a link to the multimedia resource and did not actively encourage use. It is unclear whether the intervention failed because it was not accessed, or because access to the intervention had limited impact on patient decision-making. We were unable to assess whether uptake and engagement varied across trials, or between different patient groups in individual trials.

The study was conducted pre-pandemic and the increase in the use of remote tools (including in the delivery of trials) may impact the future effectiveness of multimedia presentations in the context of trial recruitment. The creation and dissemination of the evidence was far from rapid, given recruitment began in 2012. This reflects a number of issues, including the fact that some SWATs extended beyond the funded START programme itself (hampering the completion of the meta-analysis). Some individual SWATs took a significant amount of time to complete recruitment or provide recruitment and retention data. Development of SWAT processes since that time has highlighted the need for greater efficiency, permitting faster publication of individual studies and ‘living’ meta-analyses at the level of a recruitment or retention strategy to better inform the trials community.

The participating trials were led by experienced investigators and teams, so the standard information sheets may have already been well designed, leaving less scope for improvement through intervention. To simplify ethical approvals, we compared our intervention plus standard information with standard information alone, but this may have reduced the impact of the multimedia compared to a comparison of multimedia versus standard information. Further developments in technology and media may mean that future iterations of these types of interventions could include more features and greater interactivity which might enhance effects (albeit at increased cost). All the host trials were done in the UK, making it unclear how applicable this evidence is to other countries.

### Study results in the context of the wider literature

We report here a linked series of pre-planned and co-ordinated SWATs testing the same recruitment intervention, rather than a retrospective systematic review of all relevant studies using this strategy. The studies reported here will eventually be integrated into the ongoing Cochrane review on strategies to improve trial recruitment [3], alongside similar data from studies outside the START programme. It is possible that access to multimedia has positive benefits on patient understanding, but that does not translate to improved recruitment. Assessing these sorts of impacts through a SWAT is difficult and qualitative research or process data may be required to explore such effects. It is also possible that multimedia and non-written information may be more effective for recruitment of some specific populations, for example some ethnic groups [25].

Most of the studies included in the current meta-analysis were restricted to adults. A linked study has explored the impact of multimedia on recruitment in younger populations. Data from three SWATs found that participants allocated to multimedia were more likely to be recruited to the host trial than those allocated to written information alone (OR 1.54; 95% CI 1.05, 2.28; *p* = 0.03), although multimedia did not show any impact on measures of decision-making, and the combination of multimedia and written information showed no comparative advantage [26]. Decisions about further SWAT evaluations of this technology can be based on published guidance [27], combining the results reported here, those in the TRECA study, and additional studies of this technology identified by the forthcoming Cochrane review update.

## Conclusions

A co-ordinated programme of SWATs among multiple trials found little evidence that multimedia information alongside standard information had an impact on recruitment or other outcomes.

### Supplementary Information

**Additional file 1.** Examples from the multimedia intervention.

**Additional file 2.** Presentation of the resource to patients.

**Additional file 3.** Raw data from STOP ACEI.

## Data Availability

The summary meta-analysis data is available from the corresponding author.

## Abbreviations

MRC: Medical Research Council
NRES: National Research Ethics Service
PIS(s): Participant information sheet(s)
REC: Research Ethics Committee
START: Systematic Techniques for Assisting Recruitment to Trials
SWAT: Study-within-a-trial
