# Supplementary material for: Can we achieve better trial recruitment by presenting patient information through multimedia? Meta-analysis of ‘studies within a trial’ (SWATs)
Source: BMC Med. 2023 Nov 8;21:425. doi: 10.1186/s12916-023-03081-5 (PMC10634086; doi:10.1186/s12916-023-03081-5)
Supplement: Supplementary file 1 — Additional file 1. Examples from the multimedia intervention. [file 12916_2023_3081_MOESM1_ESM.docx]

**Additional file**[**1**](https://trialsjournal.biomedcentral.com/articles/10.1186/s13063-019-3496-z#MOESM1)**Examples from the multimedia intervention**

Front screen


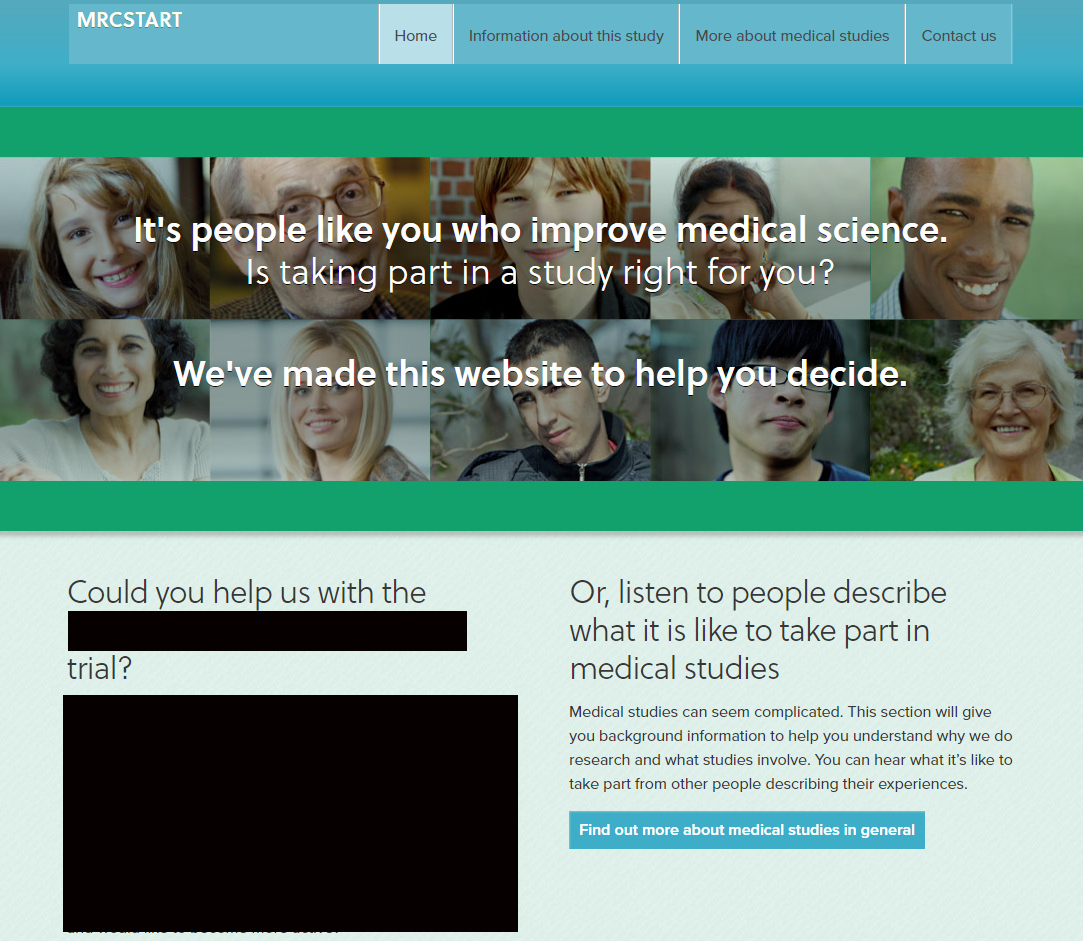


Note: The redacted areas would include details of the specific study

Study information screen


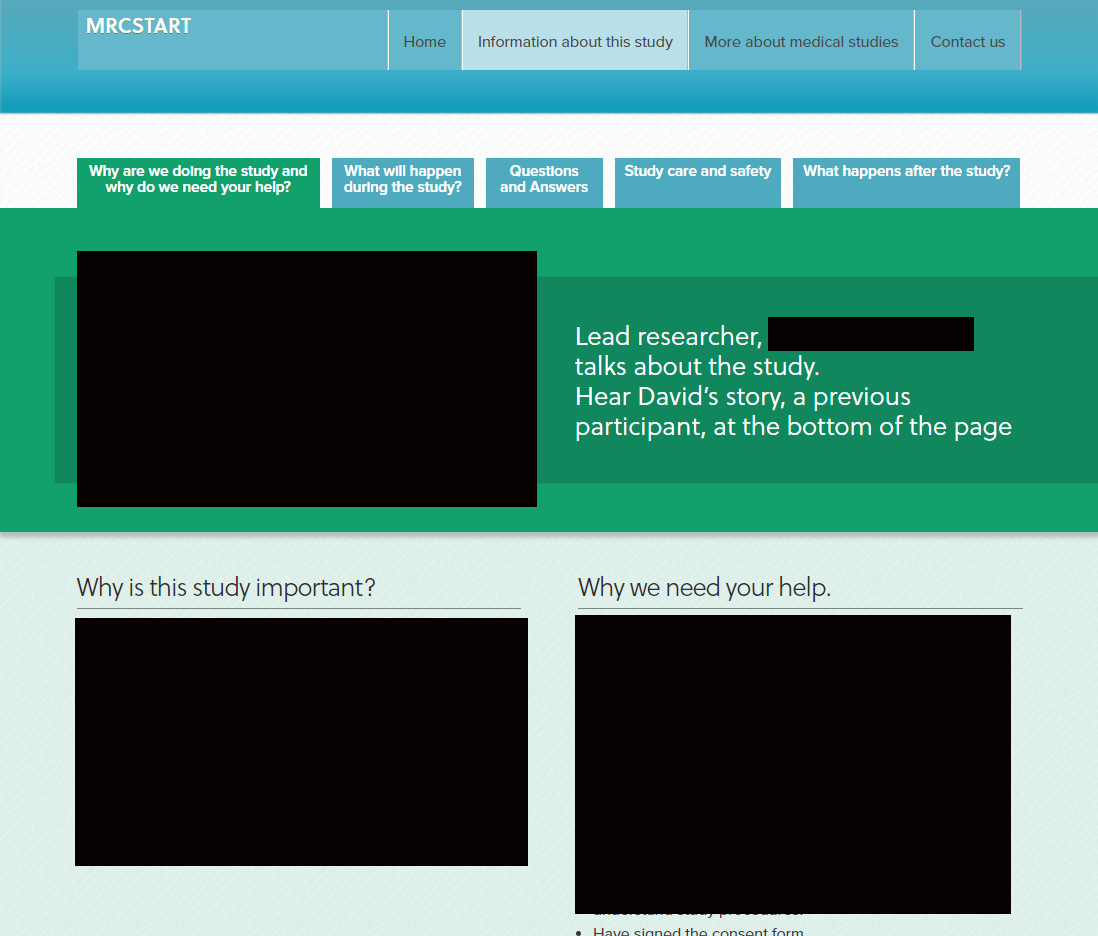


Note: The redacted areas would include details of the specific study, including a video clip from the principal investigator

Information about medical studies


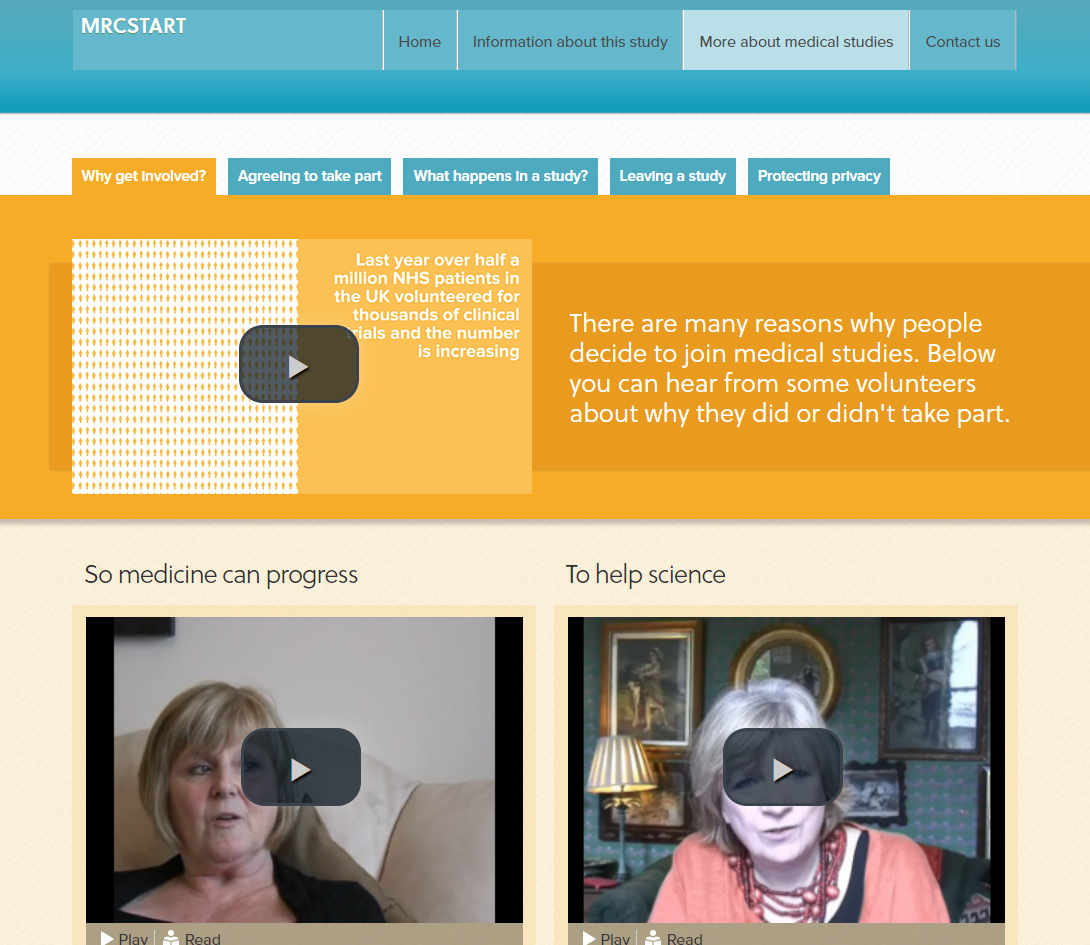


Note: The videos are from <http://www.healthtalk.org/> and are used with permission
