# Supplementary material for: Can we achieve better trial recruitment by presenting patient information through multimedia? Meta-analysis of ‘studies within a trial’ (SWATs)
Source: BMC Med. 2023 Nov 8;21:425. doi: 10.1186/s12916-023-03081-5 (PMC10634086; doi:10.1186/s12916-023-03081-5)
Supplement: Supplementary file 2 — Additional file 2. Presentation of the resource to patients. [file 12916_2023_3081_MOESM2_ESM.docx]

**Additional file**[**2**](https://trialsjournal.biomedcentral.com/articles/10.1186/s13063-019-3496-z#MOESM2)**Presentation of the resource to patients**

Date


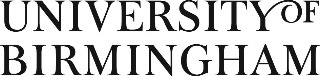
Dear ______________,

We are working with researchers from the Universities of XXX, XXX and XXX who are carrying out a study designed to investigate a method of supporting people living with chronic obstructive pulmonary disease (COPD). You may also know COPD as emphysema or chronic bronchitis. You may only experience occasional breathlessness or suffer from chest infections.

We are inviting you to take part in the study, because you **may** have COPD. The enclosed patient information letter will give you more details about the study.

However, if you have any questions, the research team at the University of XXX can be contacted on 0XX XXX XXX or by email: XXXX

If you wish to take part, please complete the enclosed reply slip and return it in the pre-paid envelope supplied.

Thank you in anticipation.

Hear from the research team at:

[www.XXXXX.com](http://www.XXXXX.com)


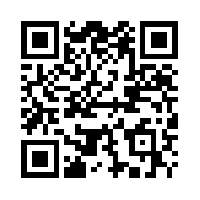


or scan the code

Yours sincerely,

______________________________________

(GP Signature)
