# Supplementary material for: Can we achieve better trial recruitment by presenting patient information through multimedia? Meta-analysis of ‘studies within a trial’ (SWATs)
Source: BMC Med. 2023 Nov 8;21:425. doi: 10.1186/s12916-023-03081-5 (PMC10634086; doi:10.1186/s12916-023-03081-5)
Supplement: Supplementary file 3 — Additional file 3. Raw data from STOP ACEI. [file 12916_2023_3081_MOESM3_ESM.docx]

**Additional file 3 Raw data from STOP ACEI**

| **Centre code** | **Allocation** | **From approach log** |  | **Accuracy of the data verified?** |
| --- | --- | --- | --- | --- |
|  |  | **Numbers approached** | **Recruited** |  |
| 66019 | MMI | 112 | 37 | No |
| 70209 | MMI | 10 | 3 | No |
| 63617 | Standard | 50 | 10 | Yes |
| 60473 | Standard | 17 | 1 | No |
| 70387 | MMI |  |  | No |
| 63822 | Standard | 38 | 8 | No |
| 57872 | MMI | 27 | 9 | Yes |
| 60891 | Standard | 36 | 10 | No |
| 60413 | Standard | 21 | 4 | No |
| 116255 | MMI |  |  | No |
| 68585 | Standard | 27 | 12 | Yes |
| 112948 | Standard | 59 | 23 | No |
| 66061 | Standard |  |  | No |
| 61211 | Standard | 9 | 5 | Yes |
| 81566 | Standard |  |  | No |
| 57329 | MMI | 23 | 13 | Yes |
| 65874 | MMI | 8 | 3 | No |
| 63250 | Standard | 24 | 0 | No |
| 61437 | MMI |  |  | No |
| 61521 | MMI | 18 | 9 | Yes |
| 66047 | MMI | 41 | 8 | Yes |
| 58358 | MMI | 14 | 5 | No |
| 78624 | Standard | 16 | 12 | Yes |
| 63450 | MMI | 9 | 2 | No |
| 62937 | MMI | 13 | 5 | Yes |
| 66541 | Standard |  |  | No |
| 57348 | MMI |  |  | No |
| 102114 | MMI | 57 | 16 | Yes |
| 57529 | Standard |  |  | No |
| 81368 | MMI | 22 | 5 | Yes |
| 139205 | Standard |  |  | No |
| 77148 | Standard |  |  | No |
| 77846 | MMI |  |  | No |
| 117249 | Standard |  |  | No |
| 109788 | Standard |  |  | No |
| 65808 | MMI |  |  | No |
| 57337 | MMI |  |  | No |
